# Toxic Elements in Traditional Kohl-Based Eye Cosmetics in Spanish and German Markets

**DOI:** 10.3390/ijerph18116109

**Published:** 2021-06-05

**Authors:** Elisabet Navarro-Tapia, Mariona Serra-Delgado, Lucía Fernández-López, Montserrat Meseguer-Gilabert, María Falcón, Giorgia Sebastiani, Sebastian Sailer, Oscar Garcia-Algar, Vicente Andreu-Fernández

**Affiliations:** 1 Grup de Recerca Infancia i Entorn (GRIE), Institut d’Investigacions Biomèdiques August Pi i Sunyer (IDIBAPS), 08036 Barcelona, Spain; elisabet.navarro@campusviu.es (E.N.-T.); gsebasti@clinic.cat (G.S.); sebastiansailer34@gmail.com (S.S.); ogarciaa@clinic.cat (O.G.-A.); 2 Department of Health, Valencian International University (VIU), 46002 Valencia, Spain; 3 Maternal & Child Health and Development Research Network-Red SAMID Health Research, Programa RETICS, Health Research Institute Carlos III, 28029 Madrid, Spain; mserrad@sjdhospitalbarcelona.org; 4 Institut de Recerca Sant Joan de Déu, 08950 Esplugues de Llobregat, Spain; 5 Departamento de Ciencias Sociosanitarias, Medicina Legal y Forense, Universidad de Murcia, 30003 Murcia, Spain; lucia.fernandez2@um.es (L.F.-L.); Montse.mg@live.com (M.M.-G.); falcon@um.es (M.F.); 6 Department of Neonatology, Hospital Clínic-Maternitat, ICGON, Institut d’Investigacions Biomèdiques August Pi i Sunyer (IDIBAPS), Barcelona Center for Maternal-Fetal and Neonatal Medicine (BCNatal), 08036 Barcelona, Spain

**Keywords:** kohl, cosmetic, toxic elements, heavy metals, lead toxicity, antimony toxicity, cadmium toxicity

## Abstract

Kohl is a traditional cosmetic widely used in Asia and Africa. In recent years, demand for kohl-based eyelids and lipsticks has increased in Europe, linked to migratory phenomena of populations from these continents. Although the European legislation prohibits the use of heavy metals in cosmetics due to the harmful effects to human health, particularly to pregnant women and children, these elements are still present in certain products. The European Union recommended levels are Pb < 20 ppm, As < 5 ppm, Cd < 5 ppm, Sb < 100 ppm, and Ni < 200 ppm. In Germany, levels are more restrictive: Pb < 2 ppm, As < 0.5 ppm, Cd < 0.1 ppm, Sb < 0.5 ppm, and Ni < 10 ppm. Here, we analyzed 12 kohl-based cosmetics in different presentations (powder, paste, and pencil) that were purchased in Spanish and German local shops. An inductively coupled plasma optical emission spectrophotometer was used to identify toxic elements and heavy metals. Levels of Pb ranged between 1.7 and 410,000 ppm in six of the study samples, four of which had levels above the recommended limit of at least two heavy metals. Arsenic (a carcinogenic element) values were within the range allowed by the EU in only 58% of the studied samples. Moreover, two products doubled this limit, reaching levels of 9.2 and 12.6 ppm. In one of the products, cadmium, related to toxic keratitis, was four times higher (20.7 ppm) than that allowed, while in two other products, these limits were doubled (11.8 and 12.7 ppm). Our results indicate the need to supervise the manufacture of kohl-based traditional products and the analysis of their composition prior distribution in European countries.

## 1. Introduction

Currently, most cosmetics are based on artificial chemicals, although there is increasing interest in products formulated on natural compounds to reduce exposure to endocrine disruptors present in many artificial goods [1]. Civilizations throughout human history have used different natural compounds to decorate their bodies [2] as part of their daily rituals [3]. Analyses of the chemical composition of these cosmetic products, as well as the consequences of their use, had not been thoroughly addressed until the mid-20th century. Some substances can enter the body through the skin, usually at lower rates than through oral ingestion or inhalation [4]. As is the case for any chemical compound introduced into the human body, some of these cosmetics show clear benefits, while others are harmful to human health [5] depending on the exposure mode, dose, and frequency of application.

Kohl (also known as surma) is a traditional cosmetic based on antimony (stibnite, Sb2S3) and used as a (medicinal) eye drop in ancient Egypt [2,6]. Over the past decades, the most common composition of kohl has been changed to galena stone, based on lead (Pb2SO4), and has become very popular in the Middle East, North and East Africa, and Asia (particularly Pakistan, Bangladesh, Nepal, India, and Iran). The popularity of kohl among these populations is not only cosmetic or cultural, but also by the believe it has therapeutic effects on eyes and as coagulant, especially in newborns and infants [7,8,9]. Antimony (Sb) is more expensive and scarcer than lead (Pb), which explains the increase in the use of the latter. In addition, Pb has been widely used by ancient physicians in the treatment of their patients, including children, and maintained over time [10]. Other components, such as carbon, titanium, or iron (gives kohl its characteristic red sheen) are also found in kohl specimens [11]. The amount and nature of the materials employed in kohl vary widely depending on where it is produced and the desired final color.

Some studies highlight the ophthalmic benefits of kohl as antimicrobial and stimulator of non-specific immunological defenses due to its ability to induce the production of nitric oxide [12]. On the other hand, there is evidence of the potential health risk of these cosmetic products due to their high content of heavy metals [13]. The human body needs certain heavy metals at specific amounts for different processes, while some elements, such as Pb or mercury, show no benefits and result in toxicity. Moreover, metals considered essential for the general homeostasis can generate toxic effects in human tissues and organs when are in excess.

Pb is mainly responsible of kohl’s toxic effects, being associated with high concentrations of this element in blood samples from regular users of this cosmetic [14,15,16]. Several studies have demonstrated that Pb levels are extremely high in this product and its use has been associated with the development of plumbism [17]. Pb is particularly harmful to the developing brain [18,19,20,21]; thus, pregnant women and children are particularly vulnerable to kohl exposure. Developmental anomalies due to prenatal and postnatal Pb exposure is correlated to reduced intellectual and learning capacities [22], as well as behavioral problems [4]. Different exposure patterns may generate distinct diseases or health problems.

The composition and the way kohl-based products are applied are factors associated to their harmful effects. These cosmetics are mixed with saliva before being applied on women’s eyebrows or the skin of children. Kohl enters the organism orally or through skin. Some authors report that <1% of Pb absorption occurs through skin, while the absorption rate of inhaled or orally ingested Pb rises to 11% in adults and 30–75% in children [23,24,25]. It is worth noting that although the rate of dermal absorption is lower, numerous pathologies have been associated to the contact with kohl. Severe corneal edema, abnormal pigmentation of the conjunctiva, and lacrimal sac and canalicular obstruction have been described [26,27].

Although Pb is the main component of kohl, other elements such as aluminum (Al) or Sb may also promote pathological dysfunctions. Exposure to Al alters neurotransmitter levels and induces choline toxicity [28,29]. Sb produces dermal and DNA strand lesions [30,31].

Immigration from regions where kohl is part of everyday life has been the route of entrance into western countries of cosmetics such as the kohl-based eyeliner. Although kohl has been banned in several countries, it can still illegally be found in some local shops worldwide [32]. The first European study evaluating the effects of kohl (1968) reported Pb-related encephalopathy in an Asian child, diagnosed in London [33]. Between 1970 and 2000, a few other studies focusing on the toxic effect of kohl were carried out in the USA and UK [34,35]. Studies analyzing the presence and effects of kohl in other European countries are scarce [36,37,38].

Although the use of certain heavy metals is prohibited in most countries, they can be present as impurities in cosmetics. There is no international agreement regarding the minimum levels of heavy metals allowed in cosmetics, and every country has its own regulations. Each country defines the legal limits and concentrations using different techniques. European Union (EU) legislation establishes its recommendations according to a risk-based approach (Pb but not kohl was banned since 1976 [39,40]), regulating that cosmetic products placed on the market in the EU cannot contain Pb, Sb, Se, or Cd, and only controlled levels of Zn. However, Germany uses technical limitations established from the element concentration in the 90th percentile after analyzing different products [4]. In the USA, kohl is not permitted in cosmetics or in any other FDA-regulated products [41].

The aim of this study was to determine the content of heavy metals in kohl products that can be purchased in Spanish and German local shops and websites to establish whether European consumers are exposed to Pb or other hazardous substances when using this type of cosmetics.

## 2. Materials and Methods

**Figure 1:**
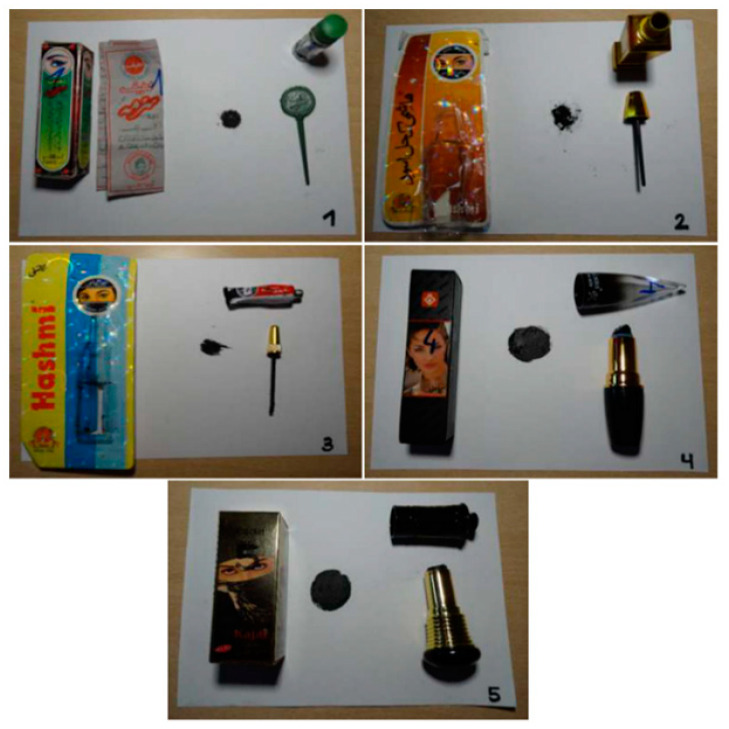
Images of kohl-based eyeliners in powder (**1** and **2**), paste (**3**), and pencil (**4** and **5**) evaluated in this study. Product labeling included cosmetic characteristics, ingredients, mode of use, application dose, precautions, and data of the person responsible. Product labeling indicated that Pb content was 0.00%.

Kohl eyeliners were purchased in person or online in Spain and Germany from 10 stores. Selection of the products was carried out until search was redundant. Twelve products with different kohl textures (powder, paste, and pencil) were included in the study for evaluation. Samples were photographed and stored at room temperature in their original packaging, coded, and specific composition registered (Figure 1). Samples 1–5 and 11 were purchased from Spain, whereas samples 6–10 and 12 were obtained in Germany.

### Analysis of Elements and Heavy Metals by Inductively Coupled Plasma Optical Emission Spectrophotometer

Element and heavy metal content of the cosmetic samples were analyzed by inductively coupled plasma optical emission spectrophotometer (ICP-OES, ICAP 6500 Duo, Thermo Scientific, UK).

First, samples were digested using 0.5 g of each kohl product and placed in 25-mL tubes with 4 mL of concentrated nitric acid and 1 mL of hydrogen peroxide 3%. Next, 300 mL of ultrapure water, 30 mL of hydrogen peroxide 33%, and 2 mL of concentrated sulfuric acid were added into the Teflon reactor. Tubes were placed in the microwave reactor with the following pressure and temperature settings: initial pressure of 40 bar, increasing pressure at 10 bar/min for 30 min and 220 °C for 20 min [42]. Samples with concentrations higher than the calibration range were diluted 1/1000.

Elements were measured at the following wavelengths (nm): As_λ_ = 193.76; B_λ_ = 208.96; Bi_λ_ = 223.07; Cd_λ_ = 214.44; Co_λ_ = 228.62; Cr_λ_ = 205.55; K_λ_ = 766.49; Li_λ_ = 670.78; Na_λ_ = 589.59; Ni_λ_ = 231.60; Pb_λ_ = 220.353; Sr_λ_ = 421.55; Tl_λ_ = 190.85; Zn_λ_ = 206.20; Rb_λ_ = 780.02. 

Elements with interference at selected wavelengths were measured at less sensitive wavelengths (Al_λ_ = 396.15; Be_λ_ = 313.04; Ca_λ_ = 315.89; Cu_λ_ = 224.70; Fe_λ_ = 238.204; Mg_λ_ = 279.08; Mn_λ_ = 259.37; Mo_λ_ = 284.82; P_λ_ = 213.7; Sb_λ_ = 206.83; Se_λ_ = 203.98; S_λ_ = 182.034; Ti_λ_ = 334.94; V_λ_ = 292.40) [42].

Each measurement was performed in triplicate (subsequent replicates) and averaged. To check for possible contamination, we analyzed 1 blank sample for every 11 samples.

For the determination of elements by inductively coupled plasma atomic emission spectroscopy we prepared multi-element calibration standards with different concentrations of inorganic elements taking as a reference the UNE-EN ISO 11885 (Table 1). In addition, intermediate standards of all elements were prepared. Table 1 shows the volumes to obtain each calibration standard and concentrations of each element in that calibration standard [42].

Equipment calibration was performed by batch and calibration established with a minimum of three points per batch. Each run was initiated with the calibration standards, followed by the samples and intermediate patterns, and the series ended with intermediate patterns (10% variation coefficient). Spike recovery tests were performed for all analyzed elements adding standard amounts of the elements to the samples before digestion, with 90–110% recoveries. Table 2 shows the detection limits and calculations of uncertainty percentages for all the elements obtained with the analytical method.

## 3. Results

We analyzed 12 kohl-based products purchased in retail shops in Spain and Germany to evaluate if their composition complies with current EU legislation on cosmetics [40], with a special focus on toxic elements such as Pb, As, Cd, and Sb [43,44]. The German legislation was also considered as reference for these analysis as it establishes more restrictive limits regarding the presence of toxic elements and heavy metals in cosmetics [45,46]. The main characteristics and information of the 12 samples analyzed is shown in Table 3. Several presentations of kohl products were evaluated (black powder, paste, or pencil), showing the ingredients on the packaging when available. Images of some of the evaluated cosmetics are shown in Figure 1. Inductively coupled plasma mass spectrometry (ICP-OES) was used to analyze the kohl samples. This technique is highly sensitive and allows for measuring multiple elements simultaneously [47,48,49].

### 3.1. Heavy Metal Content in Kohl Products

#### 3.1.1. Lead

The use of Pb and Pb-derived compounds are prohibited in cosmetics by European legislation [40] due to the harmful effects to the organism in a wide range of exposure concentrations. Regarding its toxic effects, only unavoidable impurities of Pb are allowed in cosmetic products within European borders (<20 ppm), with these limits being more restrictive in the German legislation (*BVL*, Pb < 2 ppm) [45,46].

Table 4 shows that only 3 (25%) (#5, #10, #11, blank labels) of the 12 samples evaluated meet European and German legislations. The German Federal Office of Consumer Protection and Food Safety (*BVL*) allows impurities up to 5 ppm for make-up powder, rouge, eyeshadow, eyeliner, and kajal, as well as theater and carnival make-up. Moreover, three of the other samples met European but not German rules (#4, #8, #9, orange labels), while the other six exceeded the established EU and BVL limits (#1, #2, #3, #6, #7, #12 red labels). Surprisingly, some of the values widely exceeded the limits allowed in several orders of magnitude, e.g., products #2 (122,848.82 ppm), #6 (410,806.98), and #12 (205,540.729), indicating not only that the base of these kohl products is galena (Pb2SO4), but also their potential toxic effects for the organism.

#### 3.1.2. Arsenic

Arsenic (As) is a common earth’s crust component widely distributed in tiny amounts [50,51]. As compounds are highly toxic for living organisms and have been associated to two important categories of non-melanoma skin cancers, basal cell carcinoma, and squamous cell carcinoma. As increases the generation of reactive oxidative species, aberrant immune regulations, and uncontrolled cell growth, which induces carcinogenesis. Abnormal immune activation of Langerhans and CD4^+^ cells has been showed as the potential mechanism for the deterioration of tumor control in carcinomas in chronic arsenic exposure [52]. Presence of arsenic levels considered safe in drinking water dampened the overall innate immune health in a zebrafish model [53]. This has led the EU to ban the use of As in cosmetics. Presence of As is limited to unavoidable impurities, similarly to Pb compounds (<5 ppm; <0.5 ppm in Germany). Analysis of the kohl-based study samples showed that seven products exceeded the German limitations and five the amount established by EU legislations [40] (Table 4), implying that 41.7% of the samples commercialized within European borders are illegal. As concentrations out of the recommended limits in these seven cosmetics ranged between 1.2 and 12.57 ppm, showing less variably in comparison to Pb.

#### 3.1.3. Cadmium

Cd is a known toxic compound linked to several human pathogenic processes. It is toxic at very low levels and accumulates throughout one’s lifetime. Exposure to Cd through water or food is associated with a number of illnesses including cardiovascular diseases, early atherosclerosis, kidney disease, and hypertension [54]. Although the greatest exposure to Cd occurs by tobacco smoke, there is evidence that Cd can cause severe corneal edema and toxic keratitis by contact [27]. Cd can increase endothelial cells permeability in animal models, causing corneal edema, as well as epithelial cell migration after injury [55]. Thus, the EU forbade the use of Cd and its compounds in cosmetics and only allows the presence of residual amounts establishing a legal limit of 5 ppm [40,44,46]. Cd content in three of our study samples (#2, #6, and #12) did not meet EU limitations, ranging between 11.82 and 20.75 (Table 4). When we compared the levels of our samples to the more restrictive German legislation, we found that two additional samples (#1, #3) were out of the limit, fixed in 0.1 ppm by the *BVL* [44,46].

#### 3.1.4. Antimony

Chronic exposure to Sb compounds produces a large number of adverse effects on the lungs, heart, and kidneys. Stibine is a hemolytic agent that may potentiate heart disease, pneumoconiosis, and lung cancer [56,57]. Use of Sb in cosmetics is unauthorized in the EU and its presence as an impurity restricted. Our ICP-OES results indicated that Sb levels in the 12 kohl samples were within the range <100 ppm allowed by European legislation [40], although four did not comply with the limits established by Germany (<0.5 ppm, Table 4). The low concentrations of Sb confirmed that the composition of kohl products has changed from traditional antimony (stibnite, Sb2S3) to galena stone on the basis of Pb [58,59].

#### 3.1.5. Nickel

Exposure to nickel (Ni) affects skin, causing allergic reactions. Moreover, recent studies conclude that it may be carcinogenic to humans [60]. However, the limits established for soluble Ni has remained unaltered for a decade. According to the *BVL*, Ni content must not exceed 10 ppm and up to 200 ppm according to the European legislation [40,45,46]. Analyses of the study kohl-based showed that only cosmetic #3 exceeded the German limits, indicating an adequate level for most of the products analyzed.

### 3.2. Other Metals

Use of heavy metals in cosmetics is banned in the EU (and by the *BVL*), but as previously mentioned, traces and impurities are allowed if amounts are small enough to be technically unavoidable. For a large number of heavy metals, there are no defined limits beyond representing a human health risk [40,44]. Although the presence in excess of some heavy metal may lead to health problems [55], the levels of several of these metals found in cosmetics, e.g., Cu, Fe, and Zn, are considered non-significant regarding toxicological effects in human health and are allowed in this type of product as per Regulation No. 1223/2009 of the European Parliament. Zinc oxide is considered non-toxic when used in cosmetic products [49]. Concentrations in our kohl-based products ranged between 1.98 and 360.306 mg/kg, with levels above 5000 mg/kg in 8 of the 12 cosmetics evaluated in contrast to previous studies (Table 5) [61,62]. Otherwise, Cu levels were high in the samples #2 (874.94 mg/kg), #6 (4386.50 mg/kg), and #12 (2359.97 mg/kg) [61,62]. Fe levels were lower to concentrations reported in previous studies and were within the range allowed by European law [61,62,63].

High concentrations of cobalt (Co) (used as a pigment), as well as Ni or chromium (Cr) excess, may produce allergic contact dermatitis. There is limited information on the amount of these metals that should be considered an “impurity” [64,65,66]. Impurity Cr concentration should not exceed 5 ppm, although the EU recommends that levels in color additive cosmetics should be <1 mg/kg [67]. In this study, we analyzed total Cr (Cr3^+^ plus Cr6^+^) concentrations, which must not exceed 7–10 mg/kg (U.S. Environmental Protection Agency, 1992) to avoid skin adverse effects. Cr6^+^ is a known mutagen and carcinogen and a recent meta-analysis showed that it may cause respiratory system; buccal cavity; and pharynx, prostate, and stomach cancers in humans [68]. Our results indicated that samples #1 and #3 exceed these limits. The limits established for Co are similar to those for Ni and Cr, 5 mg/kg, but the recommended limit is <1.0 mg/kg. Interestingly, only kohl-based sample #3, the same with excess of Ni and Cr, had a concentration greater than 5 mg/kg.

There is limited information on cosmetic Al toxicity due to dermal exposure [69]. A recent recommendation of the Scientific Committee on Consumer Safety (EU) indicates a threshold of 0.77% for lipsticks [70]. In our analysis, sample #3 reached a value higher than 7700 mg/kg. Manganese (Mn) is present in water and several dietary nutrients; thus, dermal contact with this metal is not generally viewed as an important risk for human health. Moreover, inorganic Mn compounds do not penetrate the skin after dermal exposure. Although excessive amounts of this metal produce manganism, a pathology with symptoms similar to those of Parkinson’s disease, there is no evidence that dermal exposure to Mn results in significant absorption through skin [70]. Inorganic selenium causes severe irritation to skin and eyes [71]. A skin sensitizer is limited in cosmetics to 1% [71]. Table 5 shows that none of the kohl samples evaluated in this study contained dangerous levels of selenium.

### 3.3. Other Elements

The EU legislation limits the use of specific Ca, B, Be, K, Li, Mg, Na, P, and S compounds but not the inorganic forms of these elements (Table 6). As a general rule, compounds containing these elements are regulated but not avoided for cosmetics manufacture [40,44,45,46]. Analysis of the kohl products evaluated here highlights the elevated values of most of these elements in samples #11 and #12 (both as powder presentations).

Finally, concentrations of other metals and elements (Table 7) in all our study samples were below the limits considered harmful for human health.

## 4. Discussion

Over the last decades, many countries have developed laws to regulate the composition of cosmetic products, with special attention to the content of heavy metals and other toxic elements. European Parliament Regulation no. 1223/2009; the Federal Food, Drug, and Cosmetic Act Chapter VI; the German *BVL*; and the Canadian Cosmetic Regulations (C.R.C., c. 869) indicate different thresholds for the amount of toxic elements and heavy metals allowed in cosmetics [40,41,46,72]. Presence in cosmetics of elements such as Pb, As, Cd, Sb, and Ni is unauthorized in all of them, only allowing their presence as trace elements derived from manufacturing processes. The primary source of metal impurities in cosmetics is the use of natural ingredients during their production. However, in some cosmetics such as kohl, the addition of mineral pigments may result in product contamination with Pb, Cd, Cr, Co, Cu, Ni, and other elements [73]. Trace amounts have to be small enough to be technically unavoidable in good manufacturing practice and safe for human health. However, differences among legislations in the limits allowed for these elements and lack of specific limits for traces that a cosmetic can contain, allowing the commercialization of traditional products manufactured with a high content of toxic elements for human health.

Kohl is one of the most used traditional cosmetics in different countries of Africa and Asia, especially by women and children. In fact, use of kohl has been detected in children under five years of age, following the advice of the family or for cosmetic reasons [74]. In some regions, it is applied to the umbilical stump of newborn babies [75]. Because of the migratory phenomena of populations from these continents to Europe over the last decades, kohl arrives through post offices, travels, or commercialization from migration origin countries to the European markets [35]. Furthermore, most exposures to this product are not detected or its risks are not considered a public health problem.

Kohl can enter the body through eyes, skin, or mouth. The toxic elements and heavy metals contained in kohl-based eye cosmetics have different absorption rates. Moreover, all heavy metals are susceptible to bioaccumulation, generating harmful effects in women and children, especially during pregnancy [76,77,78,79,80]. Studies on the effects of cosmetic and dermal Pb exposure in pregnant women are scarce. A study carried out in the Aseer (Saudi Arabia) with 176 pregnant women showed no statistically significant effects of dermal Pb exposure on pregnancy outcomes such as preterm delivery, premature rupture of the membrane, or birth weight [81]. In other studies with pregnant kohl users, Pb concentrations in blood were higher than normal levels, but lower than sub-toxic concentrations [19,82]. However, it has been emphasized that cosmetic use is a risk factor for low Pb exposure in pregnant women.

In recent years, many authors have concluded that exposure to environmental contaminants during the prenatal and perinatal periods may promote neurological and developmental impairments [83]. Pb exposure during pregnancy and childhood has been largely analyzed due to its harmful effects on development. Blood Pb levels are similar in the soon-to-be mother and the fetus because Pb can readily cross the placental barrier by simple diffusion [83]. Thus, prolonged fetal exposure to Pb due to maternal use of kohl during pregnancy may lead to adverse outcomes, including spontaneous abortion [83], low birth weight, gestational hypertension [84], and neurodevelopmental impairments [85,86]. Kohl is also commonly used as a cosmetic in children among these populations, producing mental and physical disabilities, including slowed growth, brain damage, and behavioral and learning problems [87]. The ICP-OES analysis of kohl products purchased in Spain and Germany show that half of the samples largely exceed the allowed values, with very worrying levels for human health in three of the samples (#2, #6, and #12). Our study found Pb values several orders of magnitude above the EU permitted limits (20 ppm), confirming the results of the few previous studies performed in Europe [16,43,63,88,89,90]. Our results are also in line with previous studies reporting high amounts of Pb in kohl products commercialized in America [89], Africa [17,20,32,62], and Asia [5,24,71,79,80,91,92]. Moreover, Gouitaa et al. [15] pointed out that Pb concentration of kohl products varies depending on the texture and cosmetic presentation, being higher in powder than in paste, and higher in the latter than in pencils. Our analyses confirm these differences in Pb concentrations; samples with the highest Pb concentration corresponded to kohl powders (#1, #2, #6, #7, #12), followed by kohl pastes (#3, #8), and kohl pencils (#4, #5, #9, #10), with the exception of sample #11, which is probably not based on galena.

Toxic concentrations found in our study are similar to those reported in other European studies with kohl. The range of toxic Pb concentrations in kohl described in European studies vary from 8 to 460,000 ppm [90]. Conversely, our study shows higher Pb concentrations in kohl if compared to other countries. Pb concentration in kohl found in Tunisian traditional cosmetics varies between 51.1 ppm and 4839.5 ppm [91], demonstrating lower maximum levels if compared to our results. Similarly, the content of Pb in most frequently used brands of cosmetic products in Morocco showed that Pb concentrations in kohl samples were between 0.01 and 973.8 mg/g [15]. In a study conducted in Afghanistan, 70% of the surma-based samples contained high levels of Pb (range 35–83%) [93].

With regard to other different cosmetic products used in Nigeria, Pb levels ranged from 12 to 240 ppm [62], in Pakistan 141.6 ± 0.016 ppm [5], and in Poland between 17 and 35 ppm [63]. In eye shadow samples manufactured in different countries (China, Italy, and the USA), Pb levels varied from 0.25 to 81.50 ppm [89]. These studies report low Pb levels in different cosmetics when compared to Pb levels found in kohl-based products in our study.

The above-mentioned findings show there are several kohl products available in the European markets (retail shops or internet) without accurate information on their chemical composition or the information in the packaging is missing or confusing and contain prohibited concentrations of Pb and other toxic elements.

The analysis carried out in this study shows unauthorized As levels (from 5.6 to 12.6 ppm; the limit established by the EU is 5 ppm) in samples #3, #5, #6, #11, and #12. This toxic semimetal has high affinity for skin, nails, and hair and causes adverse effects such as skin eruptions, alopecia, keratosis, and striation of the nails, and has also been associated with skin cancer and heart disease [93,94]. According to the FDA, dermal absorption of As from soil is 3%, representing dermal absorption 1% compared to ingestion [95]. According to our results, previous studies found As concentrations from 0 to 12 ppm in kohl cosmetic products [96,97,98,99,100,101,102,103,104,105,106], considered as impurities despite exceeding the established EU limits [67,89,107].

Cd toxicity after chronic exposure can cause anemia, weight loss, liver damage, and cancer. Moreover, harmful effects on steroidogenesis during pregnancy may also occur, leading to developmental impairments, suboptimal fetal growth, and spontaneous abortion [44,67,77,78,80,108]. The main route of Cd entry is by inhalation, but it can also be absorbed by ingestion and to a lesser extend through the skin [88,89]. Our ICP-OES results indicate amounts of this metal over the required limit (the EU establishes a limit of 5 ppm) in samples #2, #6, and #12 (range between 10 and 20 ppm). Cd is widely used as a color pigment in the cosmetic industry and is considered a trace element. However, several studies found prohibited amounts ranging between 0.5 and 200 ppm in different kohl-based cosmetic products in Europe [89,90] and other continents [67,79,88,89,102,103,109], in line with the results obtained for the products evaluated in this study purchased in European markets.

Sb and Ni levels are within the allowed European legislation range (100 ppm for Sb and 200 ppm for Ni), but not for the limits allowed in German regulations (0.5 and 10 ppm, respectively) where products #2, #3, #6, and #12 for Sb and #3 for Ni exceeded the allowed levels. As previously mentioned, traditional cosmetics were initially based on Sb (stibnite, Sb2S3) [6]. However, in recent years it has been replaced by galena stone (Pb_2_SO_4_), which may explain the moderate levels of Sb found in the studied products. Sb is absorbed mainly by inhalation or ingestion, but also through skin. Chronic exposure to this element produces dermatitis, Sb spots on the skin, irritation of the eyes, and gastrointestinal problems such as stomach pain and diarrhea [30,57,78,88]. Concentrations obtained in the samples of this study range between 0 and 75 ppm, in line with previous studies with kohl-based samples [56,57,79,88]. Although dermal absorption rate is 0.25% [110,111], Sb values above 5 ppm, according to German and Canadian laws, should be considered inadequate for cosmetics products. Dermal Ni absorption due to the use of kohl cosmetics produce dermatological and gastrointestinal problems [60,62,65,66,67,88]. Our results do not show high levels of this metal in our kohl-based samples, with concentrations withing the range of previous studies and far from the high values (above 100 ppm) found in some of them [14,20,62,63,67,88,89,102].

It is also important to highlight the presence of other metals and elements that may be toxic for human health, especially pregnant women and children. Co (0–8 ppm), Cr (0–20 ppm), Mn, Se, Al, and Fe levels determined in this study are in the lower limits of previous studies and are not considered dangerous for human health at low levels [14,64,65,67,88,89,90,98,112]. Surprisingly, only sample #3 shows very high levels of Co, Cr, and especially Al (12,263 ppm). Chronic exposure to Co, Cr, Cu, and Mn can cause skin problems [65,66,67,79,88,89]; some studies associate high Al exposure to neurological diseases as Alzheimer’s disease [113,114]. Moreover, the amount of Zn was elevated in 8 of the 12 cosmetics analyzed, showing higher concentrations than previous studies in Europe, Africa, and America [61,63,115]. However, Zn is considered a non-toxic compound in cosmetics [5,59].

Overall, ICP-OES analyses demonstrated that kohl-based cosmetics #2, #3, #6, and #12, purchased in Europe and representing 33% of the products evaluated in this study, contain amounts above the required levels according to EU legislation of at least two heavy metals or elements. The results of this study show that it is necessary to reinforce supervision and control measures of traditional products exported to Europe due to the high demand by African and Asian populations living in European countries. Alarmingly high levels of some highly toxic compounds, used regularly by these populations, may lead to the development of various diseases. A continuous risk communication forum might be an interesting way to bring together all aspects regarding current legislations, labeling, and toxicology. This would help industry professionals and consumers understand and communicate the risks of using this type of products. Finally, it is necessary to unify the criteria and threshold values to improve the control of heavy metal content during the manufacturing process of these cosmetic products.

## 5. Conclusions

Kohl-based cosmetics containing amounts of heavy metals above the permitted levels can be easily purchased in European markets. Moreover, information on the concentration of these metals is not indicated in the packaging or labeling of these products as established by the European legislation. The majority of the analyzed kohl samples contain prohibited concentrations of Pb, which can cause diverse harmful effects. Moreover, toxic metals as As, Cd, Sb, Ni, Zn, and Al are also present in amounts above the required limits. Our results demonstrate the need to translate this information to health professionals and review EU protocols regulating the import of cosmetic products manufactured out of Europe in order to avoid pathologies caused by heavy metals toxicity.

## Figures and Tables

**Table 1:** Concentrations of multi-element standard commercial solutions.

Name of the Standard Solution	G-0.0001	G-0.001	G-0.01	G-0.05	G-0.1	G-0.3	G-0.6	G-1.0
Volume of the Solution G-1.0 (mL)	0.01	0.1	1.0	5.0	10	30	60	100
Concentration (mL/L)								
B	0.001	0.01	0.1	0.5	1.0	3.0	6.0	10.0
Cd	0.001	0.01	0.1	0.5	1.0	3.0	6.0	10.0
Cr	0.001	0.01	0.1	0.5	1.0	3.0	6.0	10.0
Cu	0.001	0.01	0.1	0.5	1.0	3.0	6.0	10.0
Mn	0.001	0.01	0.1	0.5	1.0	3.0	6.0	10.0
Ni	0.001	0.01	0.1	0.5	1.0	3.0	6.0	10.0
Pb	0.001	0.01	0.1	0.5	1.0	3.0	6.0	10.0
Zn	0.001	0.01	0.1	0.5	1.0	3.0	6.0	10.0
As	0.001	0.01	0.1	0.5	1.0	3.0	6.0	10.0
Co	0.001	0.01	0.1	0.5	1.0	3.0	6.0	10.0
Mo	0.001	0.01	0.1	0.5	1.0	3.0	6.0	10.0
Se	0.001	0.01	0.1	0.5	1.0	3.0	6.0	10.0
Li	0.001	0.01	0.1	0.5	1.0	3.0	6.0	10.0
Sr	0.001	0.01	0.1	0.5	1.0	3.0	6.0	10.0
Rb	0.001	0.01	0.1	0.5	1.0	3.0	6.0	10.0
Tl	0.001	0.01	0.1	0.5	1.0	3.0	6.0	10.0
Sb	0.001	0.01	0.1	0.5	1.0	3.0	6.0	10.0
Ti	0.001	0.01	0.1	0.5	1.0	3.0	6.0	10.0
Be	0.001	0.01	0.1	0.5	1.0	3.0	6.0	10.0
V	0.001	0.01	0.1	0.5	1.0	3.0	6.0	10.0
Bi	0.001	0.01	0.1	0.5	1.0	3.0	6.0	10.0
Na	0.01	0.1	1.0	5.0	10	30	60	100
Mg	0.01	0.1	1.0	5.0	10	30	60	100
Al	0.01	0.1	1.0	5.0	10	30	60	100
P	0.01	0.1	1.0	5.0	10	30	60	100
S	0.01	0.1	1.0	5.0	10	30	60	100
Fe	0.01	0.1	1.0	5.0	10	30	60	100
K	0.03	0.3	3.0	15	30	90	180	300
Ca	0.1	1.0	10	50	100	300	600	1000

**Table 2:** Uncertainty of measurement (%) and detection limits for the analyzed elements by inductively coupled plasma.

Element	% Uncertainty	Detection Limit (mg/kg)	Element	% Uncertainty	Detection Limit (mg/kg)
B	3.54	0.0014	Rb	5.36	0.0012
Cd	4.56	0.0006	Sb	7.61	0.0013
Cr	4.30	0.0011	Ti	8.12	0.0014
Cu	4.12	0.0012	Be	8.91	0.0016
Mn	6.15	0.0009	V	6.78	0.0011
Ni	4.83	0.0011	Bi	5.47	0.0014
Pb	6.14	0.0008	Na	5.23	9.99
Zn	5.02	0.0014	Mg	4.70	9.85
As	5.56	0.0008	Al	5.38	0.0012
Co	5.97	0.0011	P	3.45	10.85
Mo	6.07	0.0013	S	5.24	10.05
Se	6.43	0.0012	Fe	3.92	0.0008
Li	6.78	0.0011	K	4.56	9.32
Sr	7.04	0.0010	Ca	4.34	10.23
Tl	7.05	0.0017			

**Table 3:** Checklist of the labeling in the analyzed kohl samples following the recommendations of Regulation 1223/2009, article 19 of the EU. S: Spain; G: Germany; *pw*: powder; *pt*: paste; *el*: eyeliner.

Characteristics	Kohl Samples											
	#1	#2	#3	#4	#5	#6	#7	#8	#9	#10	#11	#12
Language (English)	✗	✓	✓	✓	✓	✓	✓	✓	✓	✓	✗	✗
Company name and address	✗	✓	✗	✓	✓	✗	✓	✓	✓	✓	✗	✗
Manufacturer (country)	✗	✓	✓	✓	✓	✗	✓	✓	✓	✓	✗	✗
Content: weight or volume	✗	✓	✓	✓	✓	✓	✓	✓	✓	✓	✗	✗
Date of minimum durability	✗	✓	✓	✓	✓	✗	✓	✓	✓	✓	✗	✗
Precautions of use	✗	✗	✗	✗	✗	✗	✓	✗	✗	✗	✗	✗
Batch number	✓	✓	✓	✓	✓	✓	✓	✓	✓	✓	✓	✓
Cosmetic uses	✗	✗	✗	✗	✗	✗	✗	✗	✗	✗	✗	✗
Ingredients	✗	✓	✓	✓	✓	✗	✓	✓	✓	✓	✗	✗
Format	pw	pw	pt	el	el	pw	pw	pt	el	el	pw	pw

**Table 4:** Levels of heavy metals (*ppm* or µg/g) in kohl samples compared to the limitations established by governmental institutions. Orange boxes indicate values above the German Federal Office of Consumer Protection and Food Safety (*BVL*) [45,46] limits. Red boxes show levels which exceed the *EU* (EU) legal limits [40].

Kohl Sample	Pb (ppm)	As (ppm)	Cd (ppm)	Sb (ppm)	Ni (ppm)
**#1**	94.31	0.45	0.72	<0.0013	6.25
**#2**	122,848.82	2.78	12.75	17.59	2.07
**#3**	376.36	12.57	0.33	1.41	10.86
**#4**	7.78	1.20	0.02	<0.0013	2.43
**#5**	2.83	5.61	<0.0006	<0.0013	<0.0011
**#6**	410,806.98	9.26	20.75	75.41	2.15
**#7**	156.05	<0.0008	<0.0006	<0.0013	<0.0011
**#8**	11.78	<0.0008	0.07	<0.0013	1.24
**#9**	7.80	<0.0008	<0.0006	<0.0013	1.50
**#10**	1.73	<0.0008	<0.0006	<0.0013	<0.0011
**#11**	2.159	8.14	<0.0006	<0.0013	0.18
**#12**	205,540.73	6.18	11.82	48.38	1.68
EU limits	20	5	5	100	200
*BVL* limits	2	0.5	0.1	0.5	10

**Table 5:** Levels of other metals (*ppm* or µg/g) in kohl samples compared to the limitations established by governmental institutions. Red boxes indicate values above the German Federal Office of Consumer Protection and Food Safety (*BVL*) [45,46] and the European Union legal limits [40]. *ND*: not described.

Kohl Sample	Co (ppm)	Cr (ppm)	Cu (ppm)	Fe (ppm)	Al (ppm)	Mn (ppm)	Se (ppm)	Zn (ppm)
**#1**	0.99	5.67	9.28	4257.75	406.26	22.91	<0.0012	**9329.21**
**#2**	0.60	2.73	**874.94**	324.32	58.31	5.83	1.45	**10,717.32**
**#3**	7.64	19.62	30.04	9175.93	12,263.11	173.72	1.97	946.61
**#4**	0.15	0.00	0.56	30.43	614.89	0.71	<0.0012	**360,306.04**
**#5**	<0.0011	0.06	0.43	36.02	20.48	0.49	<0.0012	**17,002.40**
**#6**	1.32	1.44	**4386.50**	1223.45	132.26	15.17	2.48	**8428.50**
**#7**	<0.0011	<0.0011	1.63	9.57	49.43	<0.0009	<0.0012	**284,989.20**
**#8**	<0.0011	0.92	0.61	108.39	1171.73	1.34	0.15	23.44
**#9**	<0.0011	1.95	0.37	261.80	1482.30	3.04	<0.0012	428.89
**#10**	<0.0011	<0.0011	12.10	3.12	16.39	0.04	<0.0012	1.98
**#11**	<0.0011	0.28	0.577	25.95	19.599	0.725	<0.0012	**26,289.816**
**#12**	0.79	1.05	**2359.97**	984.74	79.312	8.730	2.154	**5294.492**
EU limits	5	5	ND	ND	7700	ND	10,000	ND
BVL limits	5	5	ND	ND	ND	ND	ND	ND

**Table 6:** Concentrations of elements (*ppm* or µg/g for *B*, *Be*, and *Li* and g/100 g for *Ca*, *Mg*, *K*, *Na*, *P*, and *S*) in kohl samples compared to the limitations established by governmental institutions. Orange boxes indicate values above the German Federal Office of Consumer Protection and Food Safety (*BVL*) [45,46] limits. Red boxes show levels which exceed the European Union legal limits [40]. LOD: limit of detection. *ND*: not described.

Kohl Sample	Ca (ppm)	B (ppm)	Be (ppm)	K (ppm)	Li (ppm)	Mg (ppm)	Na (ppm)	P (ppm)	S (ppm)
**#1**	0.21	310.77	<0.0016	0.090	<0.0011	0.025	0.017	192.71	2251.98
**#2**	0.03	32.55	0.44	0.003	0.14	0.003	0.001	11.32	15,637.06
**#3**	7.06	22.03	0.82	0.464	5.21	0.165	0.024	120.25	2057.33
**#4**	0.02	5.31	<0.0016	0.029	0.31	0.002	0.082	1.98	688.18
**#5**	0.018	56,235.01	<0.0016	0.002	0.52	0.005	0.023	1.25	76.82
**#6**	0.072	101.40	<0.0016	0.006	<0.0011	0.017	0.006	24.69	30,214.72
**#7**	0.005	33.24	<0.0016	0.002	<0.0011	0.001	0.001	0.58	490.71
**#8**	0.003	199.98	<0.0016	0.048	0.25	0.007	0.026	1.59	163.76
**#9**	0.003	97.53	<0.0016	0.085	0.29	0.012	0.033	<LOD	475.56
**#10**	0.004	12.47	<0.0016	<LOD	<0.0011	0.003	0.001	<LOD	45.62
**#11**	**118.15**	**117,128.61**	**<0.0016**	**23.698**	**0.689**	**29.507**	**192.067**	**15,584.94**	**882,612.18**
EU limits	ND	ND	ND	ND	ND	ND	ND	ND	ND
*BVL* limits	ND	ND	ND	ND	ND	ND	ND	ND	ND

**Table 7:** Concentrations of elements (*ppm* or µg/g) in kohl samples compared to the limitations established by governmental institutions. Orange boxes indicate values above the German Federal Office of Consumer Protection and Food Safety (*BVL*) [45,46] limits. Red boxes show levels that exceed the European Union legal limits [40]. *ND*: not described.

Kohl Sample	Bi (ppm)	Mo (ppm)	Ti (ppm)	Tl (ppm)	V (ppm)	Rb (ppm)	Sr (ppm)
**#1**	<0.0014	<0.0013	14.32	<0.0017	0.51	1.14	5.67
**#2**	118.56	0.04	0.87	16.08	0.16	0.17	9.50
**#3**	12.93	1.73	76.01	35.50	14.54	40.50	89.86
**#4**	18.16	0.05	5.15	4.44	0.17	<0.0012	1.13
**#5**	<0.0014	<0.0013	0.82	<0.0017	<0.0011	0.20	0.31
**#6**	332.59	1.83	0.94	<0.0017	0.71	0.24	56.54
**#7**	6.71	<0.0013	0.40	<0.0017	<0.0011	<0.0012	<0.001
**#8**	<0.0014	0.56	24.79	<0.0017	0.26	1.73	0.24
**#9**	<0.0014	<0.0013	57.69	<0.0017	0.17	2.60	16.80
**#10**	<0.0014	<0.0013	18.28	<0.0017	<0.0011	0.17	1.40
**#11**	2.476	0.068	0.801	<0.0017	<0.0011	0.168	0.333
**#12**	247.298	1.761	0.540	16.780	0.553	0.116	21.978
EU limits	ND	ND	ND	ND	ND	ND	ND
BVL limits	ND	ND	ND	ND	ND	ND	ND

## References

[1] Nicolopoulou-Stamati P., Hens L., Sasco A.J. (2015). Cosmetics as endocrine disruptors: Are they a health risk?. Rev. Endocr. Metab. Disord..

[2] Draelos Z.D. (2015). Cosmetics: The Medicine of Beauty.

[3] Blanco-Dávila F. (2000). Beauty and the body: The origins of cosmetics. Plast. Reconstr. Surg..

[4] Al-Dayel O., Hefne J., Al-Ajyan T. (2011). Human exposure to heavy metals from cosmetics. Orient. J. Chem..

[5] Ullah H., Noreen S., Rehman A. (2017). Comparative study of heavy metals content in cosmetic products of different countries marketed in Khyber Pakhtunkhwa, Pakistan. Arab. J. Chem..

[6] Gunn G. (2014). Critical Metals Handbook.

[7] Zaheer B.H., Mahmood Z.A., Zoha S.M.S. (1991). Therapeutic evaluation of surma (kohl) formulations. Pak. J. Sci. Ind. Res..

[8] Khan K., Saeed A., Alam M.T. (1997). Indusyunic Medicine, Traditional Medicine of Herbal, Animal and Mineral Origin in Pakistan.

[9] Pop I., Nascu C., Ionescu V., Indrea E. (1997). Structural and optical properties of PbS thin films obtained by chemical deposition. Thin Solid Films.

[10] Yaish H.M., Niazi G.A., Al Soby A. (1993). Lead poisoning among Saudi children. Ann. Saudi Med..

[11] Al-Hazzaa S.A.F., Krahn P.M. (1995). Kohl: A hazardous eyeliner. Int. Ophthalmol..

[12] Tapsoba I., Arbault S., Walter P., Amatore C. (2010). Finding out Egyptian gods’ secret using analytical chemistry: Biomedical properties of Egyptian black makeup revealed by amperometry at single cells. Anal. Chem..

[13] Bassal N., Mahmoud H.H., Fayez-Hassan M. (2013). Elemental composition study of kohl samples. Arab. J. Nucl. Sci. Appl..

[14] Al-Qutob M.A., Alatrash H.M., Abol-Ola S., Qutob M.A. (2013). Determination of different heavy metals concentrations in cosmetics purchased from the Palestinian markets by ICP/MS. AES Bioflux..

[15] Gouitaa H., Bellaouchou A., Fekhaoui M., El Abidi A., Mahnine N., Aakame R.B. (2016). Assessment of lead levels in traditional eye cosmetic “kohl” frequently used in Morocco and health hazard. J. Mater. Environ. Sci..

[16] Tiffany-Castiglioni E., Barhoumi R., Mouneimne Y. (2012). Kohl and surma eye cosmetics as significant sources of lead (Pb) exposure. J. Local Glob. Heal. Sci..

[17] Lekouch N., Sedki A., Nejmeddine A., Gamon S. (2001). Lead and traditional Moroccan pharmacopoeia. Sci. Total Environ..

[18] Awasthi S., Awasthi R., Pande V.K., Srivastav R.C., Frumkin H. (1996). Blood lead in pregnant women in the urban slums of Lucknow, India. Occup. Environ. Med..

[19] Fatmi Z., Sahito A., Ikegami A., Mizuno A., Cui X., Mise N., Takagi M., Kobayashi Y., Kayama F. (2017). Lead exposure assessment among pregnant women, newborns, and children: Case study from Karachi, Pakistan. Int. J. Environ. Res. Public Health.

[20] Nnorom I.C., Igwe J.C., Oji-Nnorom C.G. (2005). Trace metal contents of facial (make-up) cosmetics commonly used in Nigeria. Afr. J. Biotechnol..

[21] Verstraeten S.V., Aimo L., Oteiza P.I. (2008). Aluminium and lead: Molecular mechanisms of brain toxicity. Arch. Toxicol..

[22] Needleman H.L., Schell A., Bellinger D., Leviton A., Allred E.N. (1990). The long-term effects of exposure to low doses of lead in childhood. N. Engl. J. Med..

[23] Klaassen C.D. (2013). Casarett and Doull’s Toxicology: The Basic Science of Poisons.

[24] Healy M.A., Aslam M. (1989). The Asian Community: Medicines & Traditions.

[25] Aslam M., Wilson J.V. (1990). Surma—A cosmetic that can be dangerous. World Health Forum.

[26] Hidayat A., Weatherhead R.G., Al-Rajhi A., Johnson F.B. (1997). Conjunctival and lacrimal sac pigmentation by kohl (eyeliner). Br. J. Ophthalmol..

[27] Al Amry M., Al-Saikhan F., Ayoubi A. (2011). Toxic effect of cadmium found in eyeliner to the eye of a 21 year old Saudi woman: A case report. Saudi Pharm. J..

[28] Al-Saleh I., Biometals N.S. (1996). Aluminum in Saudi children. Biometals.

[29] Kumar S. (2002). Aluminium-induced changes in the rat brain serotonin system. Food Chem. Toxicol..

[30] White G.W., Mathias C.G., Davin J.S. (1993). Dermatitis in workers exposed to antimony in a melting process. J. Occup. Med..

[31] Gebel T., Birkenkamp P., Luthin S., Dunkelberg H. (1998). Arsenic (III), but not antimony (III), induces DNA-protein crosslinks. Anticancer Res..

[32] Nouioui M.A., Mahjoubi S., Ghorbel A., Yahia M.B.H., Amira D., Ghorbel H., Hedhili A. (2016). Health risk assessment of heavy metals in traditional cosmetics sold in Tunisian local markets. Int. Sch. Res. Not..

[33] Warley M.A., Blackledge P., O’gorman P. (1968). Lead poisoning from eye cosmetic. Br. Med. J..

[34] Green S., Lealman G., Aslam M., Davies S. (1979). Surma and blood lead concentrations. Public Health.

[35] Parry C., Eaton J. (1991). Kohl: A lead-hazardous eye makeup from the Third World to the First World. Environ. Health Perspect..

[36] Bruyneel M., De Caluwe J.P., Des Grottes J.M., Collart F. (2002). Use of kohl and severe lead poisoning in Brussels. Rev. Med. Bruxelles..

[37] Kervegant M., Glaizal M. (2011). Daily use of kohl at the origin of possible lead poisoning. Presse Med..

[38] Bernth N., Hansen O.C., Faergemann S., Og H., Pedersen E., Institut T. (2005). Survey of Chemical Substances in Kohl and Henna Products.

[39] Council Directive 76/768/EEC of 27 July 1976 on the approximation of the laws of the Member States relating to cosmetic products. OJEC.

[40] (2009). Regulation (EC) No 1223/2009 of the European Parlaiment of the council of 30 November 2009 on cosmetic products. https://ec.europa.eu/health/sites/default/files/endocrine_disruptors/docs/cosmetic_1223_2009_regulation_en.pdf.

[41] U.S. Food and Drug Administration Eye Cosmetic Safety. https://www.fda.gov/cosmetics/cosmetic-products/eye-cosmetic-safety.

[42] Dirección técnica Laboratorio de Ionómica CEBAS-CSIC Método de Ensayo Para la Determinación de Elementos Totales por ICP en Muestras Sólidas y Líquidas Tras Digestión Ácida. Determinación de Elementos en Muestras Líquidas por ICP. ISO 11885:1996. 2013: I. http://www.cebas.csic.es/documentos/documentos_ionomica/MET-ICP%20Rev%201.doc.

[43] Ruiz L.R., Ortiz L., Oliveira T., Abud A.M., Buchala C.A. (2019). Investigation of the presence of heavy metals and other contaminants in labor cosmetics and their health risks in general. Health Sci J..

[44] Bund B. (2017). Technically avoidable heavy metal contents in cosmetic products. J. Consum. Prot. Food Saf..

[45] Butschke A., Droß A. (2010). Die EU-kosmetikverordnung. Bundesgesundh. Gesundh. Gesundh..

[46] Goverment of Germany (2020). Berichte zur Lebensmittelsicherheit 2019 Monitoring Gemeinsamer Bericht des Bundes und der Länder.

[47] Wilschefski S.C., Baxter M.R. (2019). Inductively coupled plasma mass spectrometry: Introduction to analytical aspects. Clin. Biochem. Rev..

[48] Bua D.G., Annuario G., Albergamo A., Cicero N., Dugo G. (2016). Heavy metals in aromatic spices by inductively coupled plasma-mass spectrometry. Food Addit. Contam. Part B.

[49] Faires L.M.H. (1993). Methods of Analysis by the US Geological Survey National Water Quality Laboratory: Determination of Metals in Water by Inductively Coupled Plasma-Mass Spectrometry.

[50] WHO (2017). Arsenic. https://www.who.int/news-room/fact-sheets/detail/arsenic.

[51] Chung J.-Y., Yu S.-D., Hong Y.-S. (2014). Environmental source of arsenic exposure. J. Prev. Med. Public Health.

[52] Huang H.-W., Lee C.-H., Yu H.-S. (2019). Arsenic-induced carcinogenesis and immune dysregulation. Int. J. Environ. Res. Public Health.

[53] Nayak A.S., Lage C.R., Kim C.H. (2007). Effects of low concentrations of arsenic on the innate immune system of the zebrafish (*Danio rerio*). Toxicol. Sci..

[54] Fatima G., Raza A.M., Hadi N., Nigam N., Mahdi A.A. (2019). Cadmium in human diseases: It’s more than just a mere metal. Indian J. Clin. Biochem..

[55] Ubels J.L., Osgood T.B. (1991). Inhibition of corneal epithelial cell migration by cadmium and mercury. Bull. Environ. Contam. Toxicol..

[56] Al-Ashban R., Aslam M., Shah A. (2004). Kohl (surma): A toxic traditional eye cosmetic study in Saudi Arabia. Public Health.

[57] Andalib S., Rizwani G.H., Sharif H., Arman M. (2018). Chemical and toxicological studies on different brands of asmad (antimony sulphide) available in Pakistan and Saudi Arabia. Pak. J. Pharm. Sci..

[58] Winship K.A. (1987). Toxicity of antimony and its compounds. Advers. Drug React. Acute Poisoning Rev..

[59] Ullah P.H., Alam Mahmood Z., Sualeh M., Zoha S.M.S. (2010). Studies on the chemical composition of kohl stone by X-ray diffractometer. Pak. J. Pharm. Sci..

[60] Genchi G., Carocci A., Lauria G., Sinicropi M.S., Catalano A. (2020). Nickel: Human health and environmental toxicology. Int. J. Environ. Res. Public Health.

[61] Chauhan S.B., Chandak A., Agrawal S.S. (2014). Evaluation of heavy metals contamination in marketed lipsticks. Int. J. Adv. Res..

[62] Iwegbue C.M.A., Bassey F.I., Obi G., Tesi G.O., Martincigh B.S. (2016). Concentrations and exposure risks of some metals in facial cosmetics in Nigeria. Toxicol. Rep..

[63] Łodyga-Chruścińska E., Sykuła A., Więdłocha M. (2018). Hidden metals in several brands of lipstick and face powder present on polish market. Cosmetics.

[64] Hwang M., Yoon E.K., Kim J.Y., Son B.K., Yang S.J., Yun M.O., Choi S.S., Jang D.D., Yoo T.M. (2009). Safety assessment of chromium by exposure from cosmetic products. Arch. Pharm. Res..

[65] Basketter D.A., Angelini G., Ingber A., Kern P.S., Menné T. (2003). Nickel, chromium and cobalt in consumer products: Revisiting safe levels in the new millennium. Contact Dermat..

[66] Basketter D.A., Briatico-Vangosa G., Kaestner W., Lally C., Bontinck W.J. (1993). Nickel, cobalt and chromium in consumer products: A role in allergic contact dermatitis?. Contact Dermat..

[67] Borowska S., Brzóska M.M. (2015). Metals in cosmetics: Implications for human health. J. Appl. Toxicol..

[68] Deng Y., Wang M., Tian T., Lin S., Xu P., Zhou L., Dai C., Hao Q., Wu Y., Zhai Z. (2019). The effect of hexavalent chromium on the incidence and mortality of human cancers: A meta-analysis based on published epidemiological cohort studies. Front. Oncol..

[69] Tietz T., Lenzner A., Kolbaum A.E., Zellmer S., Riebeling C., Gürtler R., Jung C., Kappenstein O., Tentschert J., Giulbudagian M. (2019). Aggregated aluminium exposure: Risk assessment for the general population. Arch. Toxicol..

[70] Scientific Committee on Consumer Safety SCCS (2020). Opinion on the Safety of Aluminium in Cosmetic Products, Submission II.

[71] EFSA Panel on Additives and Products or Substances used in Animal Feed (FEEDAP) (2016). Safety and efficacy of selenium compounds (E8) as feed additives for all animal species: Sodium selenite, based on a dossier submitted by retorte GmbH selenium chemicals and metals. EFSA J..

[72] Goverment of Canada (2019). Cosmetic Regulations (C.R.C., C. 869). https://laws-lois.justice.gc.ca/eng/regulations/c.r.c.,_c._869/index.html.

[73] Brzóska M.M., Galazyn-Sidorczuk M., Borowska S. (2018). Metals in cosmetics. Metal Allergy: From Dermatitis to Implant and Device Failure.

[74] Mohta A. (2010). Kajal (kohl)—A dangerous cosmetic. Oman J. Ophthalmol..

[75] Darmstadt G.L., Hussein M.H., Winch P.J., Haws R.A., Lamia M., El-Said M.A., Gipson R.F., Santosham M. (2007). Neonatal home care practices in rural Egypt during the first week of life. Trop. Med. Int. Health.

[76] Wai K.M., Mar W.K., Kosaka S., Umemura M., Watanabe C. (2017). Prenatal heavy metal exposure and adverse birth outcomes in Myanmar: A birth-cohort study. Int. J. Environ. Res. Public Health.

[77] Sun H., Chen W., Wang D., Jin Y., Chen X., Xu Y. (2014). The effects of prenatal exposure to low-level cadmium, lead and selenium on birth outcomes. Chemosphere.

[78] Shirai S., Suzuki Y., Yoshinaga J., Mizumoto Y. (2010). Maternal exposure to low-level heavy metals during pregnancy and birth size. J. Environ. Sci. Health Part A.

[79] Zheng G., Zhong H., Guo Z., Wu Z., Zhang H., Wang C., Zhou Y., Zuo Z. (2014). Levels of heavy metals and trace elements in umbilical cord blood and the risk of adverse pregnancy outcomes: A population-based study. Biol. Trace Elem. Res..

[80] CChen Z., Myers R.P., Wei T., Bind E., Kassim P., Wang G., Ji Y., Hong X., Caruso D., Bartell T. (2014). Placental transfer and concentrations of cadmium, mercury, lead, and selenium in mothers, newborns, and young children. J. Expo. Sci. Environ. Epidemiol..

[81] Mirghani Z. (2010). Effect of low lead exposure on gestational age, birth weight and premature rupture of the membrane. J. Pak. Med. Assoc..

[82] Moghraby S., Abdullah M., Karrar O., Akiel A., Shawaf T., Majid Y. (1989). Lead concentrations in maternal and cord blood in women users of surma eye cosmetics. Ann. Trop. Paediatr..

[83] Falcon M. (2003). Placental lead and outcome of pregnancy. Toxicology.

[84] Magri J., Sammut M., Savona-Ventura C. (2003). Lead and other metals in gestational hypertension. Int. J. Gynecol. Obstet..

[85] Shen X.-M., Yan C.-H., Guo D., Wu S.-M., Li R.-Q., Huang H., Ao L.-M., Zhou J.-D., Hong Z.-Y., Xu J.-D. (1998). Low-level prenatal lead exposure and neurobehavioral development of children in the first year of life: A prospective study in Shanghai. Environ. Res..

[86] Wasserman G., Liu X., Popovac D., Factor-Litvak P., Kline J., Waternaux C., LoIacono N., Graziano J. (2000). The Yugoslavia prospective lead study: Contributions of prenatal and postnatal lead exposure to early intelligence. Neurotoxicol. Teratol..

[87] Hauptman M., Bruccoleri R., Woolf A.D. (2017). An update on childhood lead poisoning. Clin. Pediatr. Emerg. Med..

[88] Bocca B., Pino A., Alimonti A., Forte G. (2014). Toxic metals contained in cosmetics: A status report. Regul. Toxicol. Pharmacol..

[89] Volpe M., Nazzaro M., Coppola R., Rapuano F., Aquino R.P. (2012). Determination and assessments of selected heavy metals in eye shadow cosmetics from China, Italy, and USA. Microchem. J..

[90] Filella M., Martignier A., Turner A. (2020). Kohl containing lead (and other toxic elements) is widely available in Europe. Environ. Res..

[91] Mehrpour O., Karrari P., Abdollahi M. (2012). Chronic lead poisoning in Iran; a silent disease. DARU J. Pharm. Sci..

[92] McMichael J.R., Stoff B.K. (2017). Surma eye cosmetic in Afghanistan: A potential source of lead toxicity in children. Eur. J. Nucl. Med. Mol. Imaging.

[93] Tseng C.-H., Chong C.-K., Tseng C.-P., Hsueh Y.-M., Chiou H.-Y., Chen C.-J. (2003). Long-term arsenic exposure and ischemic heart disease in arseniasis-hyperendemic villages in Taiwan. Toxicol. Lett..

[94] Naujokas M.F., Anderson B., Ahsan H., Aposhian H.V., Graziano J.H., Thompson C., Suk W.A. (2013). The broad scope of health effects from chronic arsenic exposure: Update on a worldwide public health problem. Environ. Health Perspect..

[95] U.S. Environmental Protection Agency (2004). Risk Assessment Guidance for Superfund (RAGS): Part E | Risk Assessment.

[96] Faruruwa M.D., Bartholomew S.P. (2014). Study of heavy metals content in facial cosmetics obtained from open markets and superstores within Kaduna metropolis, Nigeria. Am. J. Chem. Appl..

[97] Umar M.A., Caleb H. (2013). Analysis of metals in some cosmetic products in FCT-Abuja, Nigeria. Int. J. Res. Cosmet. Sci..

[98] Nico P.S., Ruby M.V., Lowney Y.W., Holm S.E. (2006). Chemical speciation and bioaccessibility of arsenic and chromium in chromated copper arsenate-treated wood and soils. Environ. Sci. Technol..

[99] Rahman M., Vahter M., Wahed M.A., Sohel N., Yunus M., Streatfield P.K., El Arifeen S., Bhuiya A., Zaman K., Chowdhury A.M.R. (2006). Prevalence of arsenic exposure and skin lesions. A population based survey in Matlab, Bangladesh. J. Epidemiol. Commun. Health.

[100] Kulwa G.S., Mihale M.J. (2020). Levels and exposure risks of lead, arsenic and mercury from selected lipstick and nail polish cosmetics marketed in Dar es Salaam, Tanzania. Tanzan. J. Sci..

[101] Contado C., Pagnoni A. (2012). A new strategy for pressed powder eye shadow analysis: Allergenic metal ion content and particle size distribution. Sci. Total Environ..

[102] Omolaoye J., Uzairu A., Gimba C.E. (2010). Heavy metal assessment of some eye shadow products imported into Nigeria from China. Arch. Appl. Sci. Res..

[103] Adepoju-Bello A.A., Oguntibeju O.O., Adebisi R.A., Okpala N., Coker H.A.B. (2012). Evaluation of the concentration of toxic metals in cosmetic products in Nigeria. Afr. J. Biotechnol..

[104] Peter O.O., Eneji I.S., Sha’Ato R. (2012). Analysis of heavy metals in human hair using atomic absorption spectrometry (AAS). Am. J. Anal. Chem..

[105] Sainio E.-L., Jolanki R., Hakala E., Kanerva L. (2000). Metals and arsenic in eye shadows. Contact Dermat..

[106] Mohammed T.I., Mohammed E., Bascombe S. (2017). The evaluation of total mercury and arsenic in skin bleaching creams commonly used in Trinidad and Tobago and their potential risk to the people of the Caribbean. J. Public Health Res..

[107] Pinol S., Sala A., Guzman C., Marcos S., Joya X., Puig C., Velasco M., Vélez D., Vall O., Garcia-Algar O. (2015). Arsenic levels in immigrant children from countries at risk of consuming arsenic polluted water compared to children from Barcelona. Environ. Monit. Assess..

[108] Stasenko S., Bradford E.M., Piasek M., Henson M.C., Varnai V.M., Jurasović J., Kusšc V. (2010). Metals in human placenta: Focus on the effects of cadmium on steroid hormones and leptin. J. Appl. Toxicol..

[109] Nourmoradi H., Foroghi M., Farhadkhani M., Dastjerdi M.V. (2013). Assessment of lead and cadmium levels in frequently used cosmetic products in Iran. J. Environ. Public Health.

[110] Roperm S.C., Stupart L. (2006). The In Vitro Percutaneous Absorption of Antimony Trioxide Through Human Skin.

[111] Centre for Public Health Research (2011). Report for a Survey of Low Cost Cosmetics (Lipstick) for the Ministry of Health.

[112] Kang E.K., Lee S., Park J.-H., Joo K.-M., Jeong H.-J., Chang I.S. (2006). Determination of hexavalent chromium in cosmetic products by ion chromatography and postcolumn derivatization. Contact Dermat..

[113] Campbell A. (2002). The potential role of aluminium in Alzheimer’s disease. Nephrol. Dial. Transplant..

[114] Exley C. (2016). The toxicity of aluminium in humans. Morphologie.

[115] De Souza Castro C.F., Brandão R.R., Pescara I.C., Toscano I.A.S., Zara L.F. (2010). Heavy metals determination in brazilian lipsticks. Glob. Sci. Technol..

